# Statement of Retraction: Circ 003390/Eukaryotic translation initiation factor 4A3 promoted cell migration and proliferation in endometrial cancer via vascular endothelial growth factor signaling by miR-195-5p

**DOI:** 10.1080/21655979.2026.2729943

**Published:** 2026-10-01

We, the Editors and Publisher of the journal *Bioengineered*, have retracted the following article from publication.

Ma, J., Zhao, X., & Shi, L. (2022). Circ 003390/Eukaryotic translation initiation factor 4A3 promoted cell migration and proliferation in endometrial cancer via vascular endothelial growth factor signaling by miR-195-5p. *Bioengineered*, 13(5), 11958–11972. https://doi.org/10.1080/21655979.2022.2069358

Since publication, significant concerns have been raised about the integrity of the data and reported results in the article. These concerns include alleged image duplication with a similar image from an unrelated article

•     Figure 4i (circWEE1+si-EIF4A3), appears to have been duplicated with a similar image in Figure 7C (LPS+ATP+SCH) from Zhang et al, 2021 (https://doi.org/10.18632/aging.203611)

When approached for an explanation, the authors did not respond to our queries and so these serious concerns remain unaddressed.

As verifying the validity of published work is core to the integrity of the scholarly record, we are therefore retracting the article. The authors have been informed of this decision.

We have been informed in our decision-making by our policy on publishing ethics and integrity and COPE guidelines. 

The retracted article will remain online to maintain the scholarly record and will be digitally watermarked on each page as ‘Retracted’. 

